# Simulation of the Particle Transport Behaviors in Nanoporous Matter

**DOI:** 10.3390/polym14173563

**Published:** 2022-08-29

**Authors:** You Wu, Dandan Ju, Hao Wang, Chengyue Sun, Yiyong Wu, Zhengli Cao, Oleg V Tolochko

**Affiliations:** 1 School of Material Science and Engineering, Harbin Institute of Technology, Harbin 150006, China; 2 Xi’an Aerospace Chemical Propulsion Co., Ltd., Xi’an 710000, China; 3 Research Center of Space Physics and Science, Harbin Institute of Technology, Harbin 150006, China; 4 Aerospace System Engineering Shanghai, Shanghai 200000, China; 5 School of Material Science and Engineering, Peter the Great Saint Petersburg Polytechnic University, 195251 Saint Petersburg, Russia

**Keywords:** Monte Carlo, nanoporous matter, proton, transport behavior, GEANT4 code

## Abstract

The transport behaviors of proton into nanoporous materials were investigated using different Monte Carlo simulation codes such as GEANT4, Deeper and SRIM. The results indicated that porous structure could enhance the proton scattering effects due to a higher specific surface area and more boundaries. The existence of voids can deepen and widen the proton distribution in the targets due to relatively lower apparent density. Thus, the incident protons would transport deeper and form a wider Bragg peak in the end of the range, as the target materials are in a higher porosity state and/or have a larger pore size. The existence of voids also causes the local inhomogeneity of proton/energy distribution in micro/nano scales. As compared, the commonly used SRIM code can only be used to estimate roughly the incident proton range in nanoporous materials, based on a homogeneous apparent density equivalence rule. Moreover, the estimated errors of the proton range tend to increase with the porosity. The Deeper code (designed for evaluation of radiation effects of nuclear materials) can be used to simulate the transport behaviors of protons or heavy ions in a real porous material with porosity smaller than 52.3% due to its modeling difficulty, while the GEANT4 code has shown advantages in that it is suitable and has been proven to simulate proton transportation in nanoporous materials with porosity in its full range of 0~100%. The GEANT4 simulation results are proved consistent with the experimental data, implying compatibility to deal with ion transportation into homogeneously nanoporous materials.

## 1. Introduction

With the development of nuclear energy and space exploration, materials with excellent irradiation tolerance are receiving extensive attention [1,2]. Numerous effects have been devoted to investigate the damage behaviors and mechanisms of nuclear protection materials under various irradiation resource [3,4,5,6,7]. For metals or inorganic crystal materials, energetic particle radiation was found to cause point defects, and the accumulation of point defects would form dislocation loops, voids, stacking fault tetrahedrons (SFTs), etc. [8]. The key to design the radiation tolerant materials is that the point defects can be partly recovered or eliminated during the radiation process [1,9]. The most popular approach is to introduce interfaces inside the materials [1,9,10], using a free surface to attract, absorb and annihilate point and line defects [7,10,11]. Chen [12] investigated the radiation response and mechanisms in a helium ion-irradiation immiscible coherent Cu/Co nanolayer and found that the interface of Cu/Co can effectively reduce the population of irradiation-induced defects.

Recently, owing to its lightweight, ultra-low metal consumption and large free surface area per unit volume [10,13], nanoporous (NP) metal shows great potential to become a new class of radiation-resistant material. Bringa [9] reported the existence of a radiation-resistant window between the ligament size of NP foams and the irradiation dose rate. Li [10] studied the irradiation response of nanoporous Au under heavy ion beam bombardment in situ and indicated that the radiation-induced defects can be absorbed/eliminated by nanopores.

To date, the previous research has demonstrated that NP metals can be reasonably designed with strength, stability and radiation resistance [9,14], but there is still a lack of corresponding simulation calculation about the ion transport behavior in nanoporous matter (metal, inorganic and polymer). Compared to nanoporous metal and inorganic materials, nanoporous polymers exhibit excellent flexibility, thermal insulation and stability and have received extensive attentions recently [15]. Owing to their special structure (large specific surface area and high porosity) and ultra-low density, which make them an ideal material for some specific fields, such as filtration [16], catalysis and wastewater treatment field [17,18,19], as well as the thermal/sound insulation and aerospace activity fields [20]. The stability of a nanoporous polymer must be considered under space irradiation environments. On-orbit experiments and ground-based irradiation simulation tests are expensive and time-consuming [21], while computer simulation is a common and reliable method for evaluating the radiation effects of materials. Porosity and pore size are the main structural parameters of porous materials. However, what is not yet known is how porosity and pore size affect the transport process. In the previous research, Stopping and Range of Ions in Matter (SRIM) software [22] is always used to estimate the range of charged particles into porous matter [9]. However, SRIM can only be used to handle homogeneous or multiple-layered substances, nevertheless, it would fail in dealing with the transport behavior of ions into heterogeneous matter. It is essential to model nanoporous matter and understand the transport behavior of charged particles into nanoporous matter.

In this work, the study is divided into two sections. The first section is to simulate the transport behavior of a proton into nanoporous matter, to make clear whether the porous part takes special roles on the transport process (range, particle distribution and energy deposition). The second section is to investigate the damage behaviors of nanoporous matter (using polyimide aerogel as an example) under proton irradiation, to compare with/verify the simulation results. The obtained results for transport behaviors in porous matter would provide a path to future complex simulations calculation and irradiation-resistant nanoporous materials design.

## 2. Materials and Methods

### 2.1. Simulation Codes

The software packages of SRIM (United States) are based on Monte Carlo method [22], which is commonly used to simulate the interaction of ion beam with homogeneous or quasi-homogeneous matters (such as laminated materials).

Deeper [23] (DamagE crEation and Particle transport in matter, China) is another code based on Monte Carlo method, which can only be used to handle porous materials transport process with porosity less than 52.3%.

GEANT4 [24] is a Monte Carlo application toolkit developed by CERN (European Organization for Nuclear Research, Geneva, Switzerland), which is used to simulate the physical process of particle transport in matter. In this work, Deeper and GEANT4 were used to simulate the proton into nanoporous materials.

### 2.2. Proton Irradiation

The irradiation experiments were carried out using a ground simulator of space particle-radiation environments in Harbin Institute of Technology. This irradiation equipment can simulate proton, electron and ultraviolet radiation. The energy of proton and electron can be set from 30 to 200 keV, and the equipment details are described elsewhere [25]. In this work, the proton energy was set as 170 keV at a flux at 5 × 10^11^ cm^−2^s^−1^. The proton fluence was set up to 5 × 10^14^ cm^−2^ in the tests. During the irradiation, the proton beam was incidented perpendicularl into the samples surface with scanning area of 10 × 10 mm. The tested chamber was kept in a vacuum better than 10^−5^ Pa.

### 2.3. Materials and Testing

**Figure 1:**
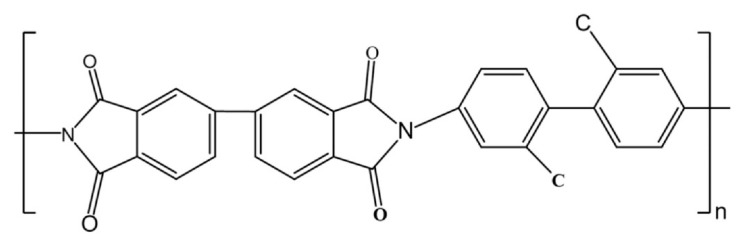
The chemical structure of the repeating unit of the PI aerogel.

In this work, the polyimide aerogels were obtained from Aerospace System Engineering (Shanghai, China), with specimen size 10 × 10 × 5 mm. The synthesis detail is described elsewhere [26], and the chemical structure is shown in Figure 1. The porosity of the pristine samples is 94%, and the skeleton density of polyimide is 1.4 g·cm^−3^, which is obtained using Helium pycnometer. The average pore size is measured as a diameter of 51 nm using 3H-2000PS2 nitrogen isotherm adsorption instruments, and the specific surface area is 293 m^2^/g calculated by BET (Brunauer–Emmett–Teller) model. After the irradiation, the samples were notched from the non-irradiated side and fractured. The samples were coated with a platinum film for conductivity before observation, then A ZEISS field emission scanning electron microscopy from Merlin Compact was used to investigate the sample cross section. The acceleration voltage is 5 keV, and the working distance is 8 mm during tests.

## 3. Results and Discussion

### 3.1. Simulation Part

#### 3.1.1. Porosity Effects

**Figure 2:**
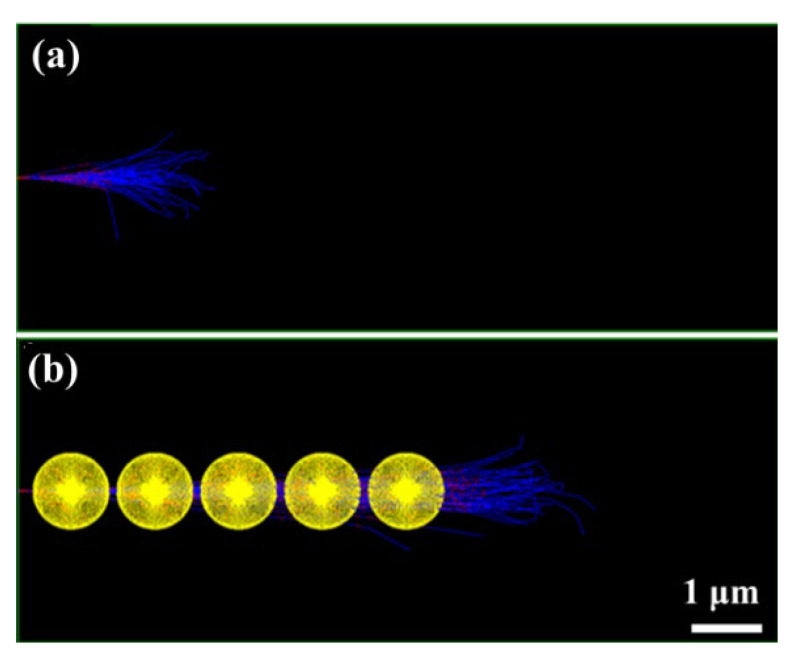
The transport behavior calculated by GEANT4: (**a**) Solid. (**b**) Geometric structure containing voids. (The black box represents the solid materials; the yellow balls represent the voids; the blue lines represent the ion tracks.).

Figure 2 shows the comparison of the incident proton tracks in a solid and corresponding geometric structure with voids. It can be seen that the apparent range of the incident protons is seen much deeper and more scattered in the porous matter, implying that large number of nano-voids in the materials may exert drastic change (such as proton distribution, energy deposition, etc.) as compared with that in a normal solid matter.

**Figure 3:**
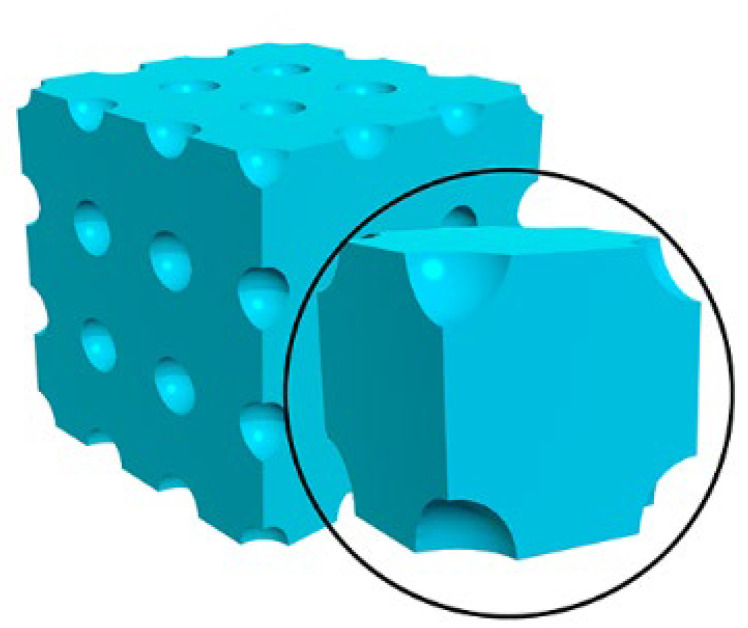
The schematic diagram of spherical periodic porous structure.

In order to better understand the transport behavior of proton into nanoporous matter, a simple model was proposed for simulating the particle transport parameter in nanoporous solids, as shown in Figure 3.

Given that the nano-porous materials are composed of spherical pores homogeneously distributed in a solid, the porous materials could be modeled as a continuous solid including periodic distributed spherical nano-holes or voids. The spacing of the adjacent voids in the coordination axes of *X*, *Y* and *Z* could be defined as *a*, *b* and *c*, respectively. The radius of voids is set as *r*. In this case, the incident particles (such as protons) should transport straightly within the voids. According to the above-defined scaling parameters, the porosity of the defined materials can be calculated using Formula (1):

(1) $$\eta =\frac{4\pi r^{3}}{3abc}$$

**Figure 4:**
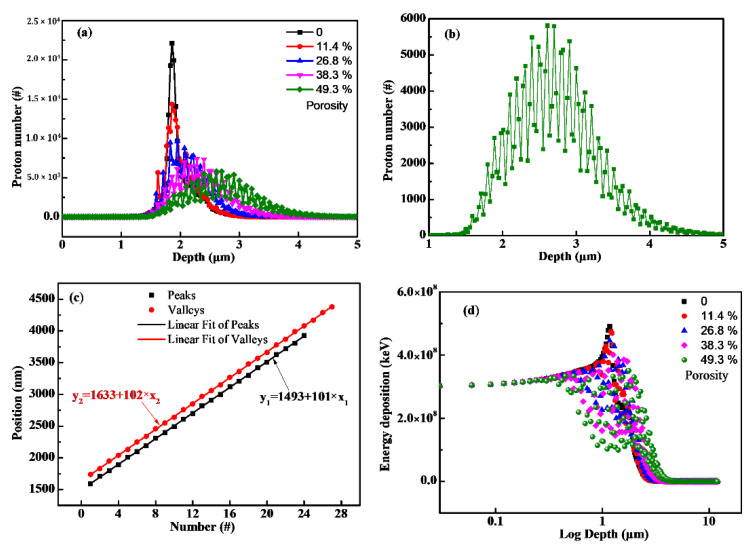
(**a**) The relationship between proton distribution and porosity (the radius of the voids was set as 50 nm, and the distance between the adjacent voids was set as 102 nm, 111 nm, 125 nm and 166 nm, respectively); (**b**) a zoom view of 49.3% porosity in (**a**); (**c**) the position of peaks and valleys as a function of number; (**d**) the relationship between energy deposition and porosity.

Based on the periodic nanoporous model, the transport behavior of proton into materials can be calculated using GEANT4 code. Figure 4a shows the simulated results of protons incident into different porosity materials. It can be seen that increasing the porosity, the proton range was observed increase together with a broadened Bragg peak. Moreover, one could also observe multiple subpeaks overlying on the Bragg peak. In order to analyze this phenomenon, the proton distribution curves at the porosity of 49.3% were enlarged, as shown in Figure 4b. It is worth noting that the overlying subpeaks appear periodically, and the periodic distance between adjacent sub-peaks is about 102 nm, the same as that of the adjacent voids. To better understand this phenomenon, the peak/valley positions and the corresponding numbers were counted, as shown in Figure 4c. Interestingly, there is an obvious linear relationship between the position and number of these subpeaks/valleys. The fitting slope of peaks and valleys is almost equivalent to the distance of adjacent voids (102 nm). In addition, the intercept represents the first apparent subpeak or subvalley in the distribution curves. In addition, the intercept of the peaks linear fitting equal to 14.6 times the adjacent void’s distance (102 nm), while the intercept of the valleys is equal to 16 times the adjacent void’s distance (102 nm). The results indicate the existence of voids would cause the local inhomogeneity of proton distribution. More specifically, there are fewer protons deposited at the center position (namely, subvalleys position) of the spherical voids, and more protons deposited at the right of the spherical void’s edge (namely, subpeaks position). Figure 4d depicts the relationship between energy deposition distribution and porosity. It shows similar characteristic to the proton distributions in the porous materials, namely energy deposition, tend to be deeper and broader in the materials when increasing the porosity. The results indicate that the existence of voids would magnify the transport process of protons and cause local inhomogeneity around the spherical voids.

**Figure 5:**
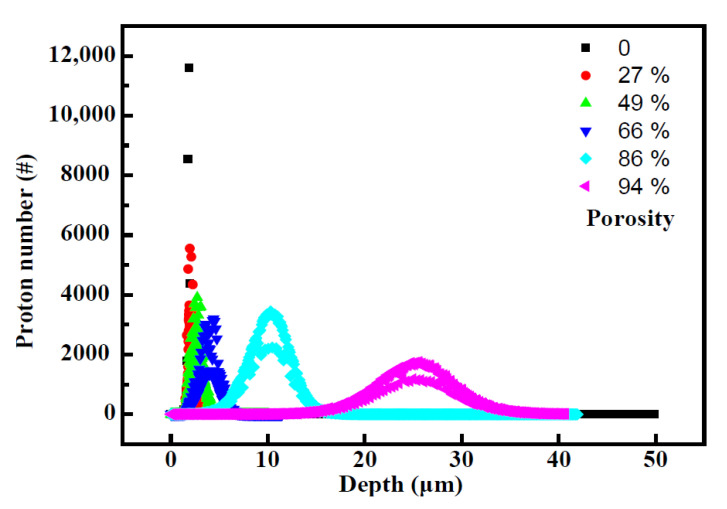
The relationship between porosity and proton distribution (the voids were set as a 100 nm cube, and the distance between the adjacent voids was set as 102 nm, 105 nm, 115 nm, 126.6 nm and 155 nm, respectively).

However, due to the limitation of the geometric structure (2 × r < a), the porosity of this periodic model cannot exceed 52.3%. In this case, as the spherical pores are replaced by cubic ones, for modeling the porous materials, the porosity of the porous matter can vary from 0% to 100%. Figure 5 shows the relationship between proton distribution and porosity ranged from 0%~100%. One can see similar behaviors of proton incidents, into the materials with various porosities as the above simulated results, namely larger ranges, apparently broaden Bragg peak with overlying subpeaks and lower intensity in the end of the range.

As comparison, the software of SRIM was also applied to evaluate the proton transport behavior in the porous materials, only if one assumes the porous materials as homogeneous, with lower density according to the porosity. Hence, the porous matter can be homogenized through Formula (2) and then the apparent density could be used for the SRIM simulation.

(2) $$\rho =(1-\eta )\cdot \rho_{0}$$

where *ρ*_0_ is the density of the skeleton medium, *ρ* is the apparent density after homogenization and *η* is the porosity.

**Figure 6:**
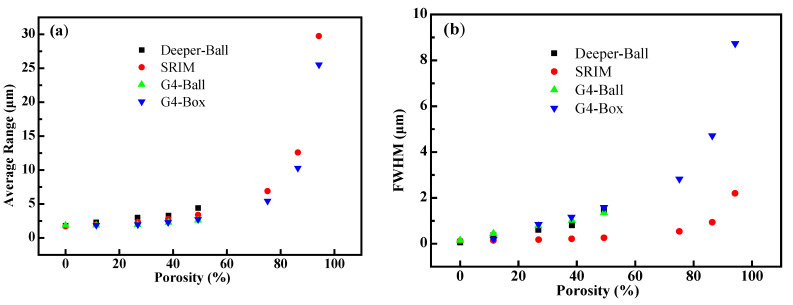
The transport results calculated by different software (G4-Ball represents the GEANT4 simulation results with spherical voids, while the G4-Box represents the GEANT4 simulation results with cubic ones, and Deeper-ball represents the Deeper simulation results with spherical voids): (**a**) the relationship between average range and porosity; (**b**) the relationship between FWHM and porosity.

Figure 6 shows the simulation results calculated using different codes. It is understandable that for three cases simulated using GEANT4, Deeper and SRIM codes, the average proton ranges increase with the porosity in that the apparent density of the porous matter decreases accordingly (Figure 6a). However, the results of GEANT4/Deeper show some differences from those obtained using SRIM code. On one hand, the proton ranges simulated by any of the three codes (GEANT4, Deeper and SRIM) are almost the same as the porosity is smaller (Figure 6a), but when the porosity is larger than 50%, the calculated range using GEANT4 is smaller than that of SRIM. Hence, when the porosity is as large as 94%, the ranges calculated by GEANT4 and SRIM were 25.5 and 29.8 μm, respectively. It is worth noting that this deviation becomes more pronounced as the porosity increases (Figure 6a). However, the FWHM (full width at half maxima) of the Bragg peak calculated by GEANT4 with cube voids is about 9 μm at the porosity of 94%, while the FWHM is about 2 μm using SRIM (Figure 6b). It can be concluded that existence of voids (GEANT4/Deeper) in solid causes more broaden distribution of incident particles than the density of the homogenized porous matter (SRIM), since that inhomogeneity in matter could enhance the scattering processes of the incident particle. When the porosity is less than 50%, SRIM [22] can be used to calculate the range of proton into porous matter, but when porosity increases, it is necessary to use GEANT4 [24] to deal with it.

#### 3.1.2. Voids Size Effect

**Figure 7:**
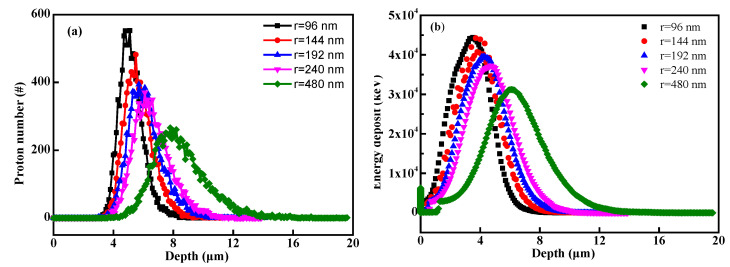
(**a**) The relationship between proton distribution and pore size (the porosity was set as 46.3%, and the radius was set as 96, 144, 192, 240 and 480 nm, respectively); (**b**) the relationship between energy deposition and pore size.

In order to further explore the voids size effects on the transport process, the proton distribution was calculated while the porosity remains the same, and the results are shown in Figure 7. Interestingly, the distribution of protons became broader, while the range deepened, when increasing the pore radius, even though the porosity is same. Correspondingly, energy deposition in the porous matter distributes more broadly and tends to have deeper positions. The results indicated that the pore size would also magnify the transport straggling process of ion beam in matter, due to the coupling behavior between the scattering effects from the porous boundaries and the longer linear-transporting characteristic within the larger void.

**Figure 8:**
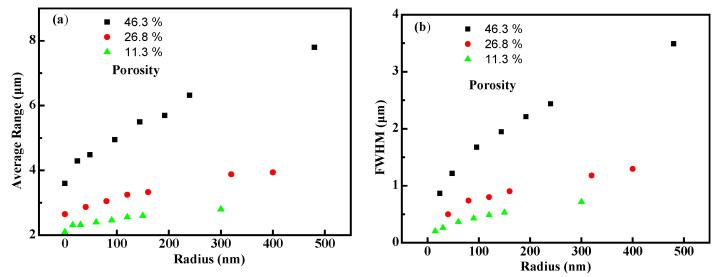
(**a**) The relationship between proton range and radius of voids (when the porosity was kept at 46.3%, the radius was set as 24, 48, 96, 144, 192, 240 and 480 nm, respectively; when the porosity was kept at 26.8%, the radius was set as 40, 80, 120, 160, 320 and 400 nm, respectively; when the porosity was kept at 11.3%, the radius was set as 15, 30, 60, 90, 120, 150 and 300 nm, respectively); (**b**) the relationship between FWHM and radius of voids.

Figure 8 depicts the change rules of the proton range and the FWHM of Bragg peak as functions of the porous size. One can see that the proton range (Figure 8a) and FWHM (Figure 8b) increase with void radius without changing the porosity. On the other hand, there is an important parameter, called specific area, for a porous matter to define its performances. As we know, smaller void size means a larger specific surface area, as the matter porosity is constant [27]. Thus, the results shown in Figure 8 imply that with a constant porosity, the smaller the void size, the larger the specific surface area, and then the proton range and also the FWHM of its Bragg peak tend to be smaller. It is reasonable to say that the larger a specific surface area is, the more opportunities for the incident protons to interact with the surface/boundary and the higher the possibilities to be scattered and stopped [27], thus, a smaller proton average range and FWHM can be obtained.

### 3.2. Experiments Part

**Figure 9:**
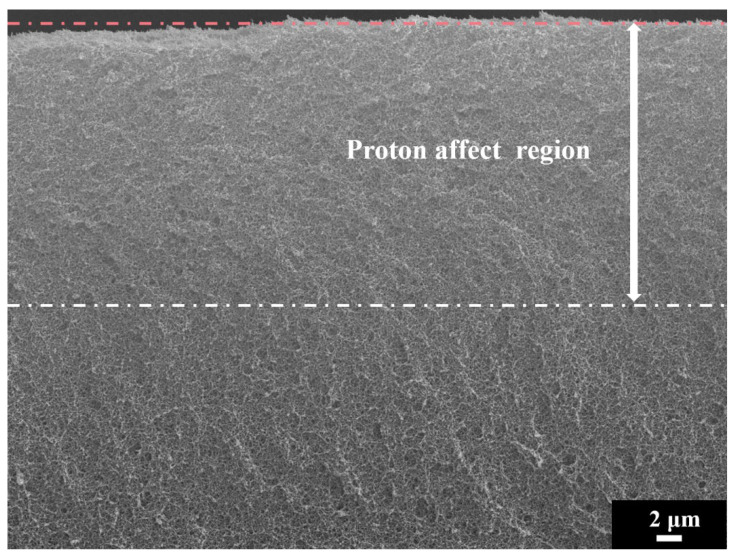
SEM cross-section images of polyimide aerogels after proton irradiations at fluences of 5 × 10^14^ cm^−2^ (the orange dashed line is the upper edge of the sample’ section, and the white dashed line is the boundary between the damaged area and damage-free area.).

In order to verify the simulated results, a proton irradiation experiment was carried out. Figure 9 depicts the SEM cross-section images of the polyimide aerogels (with a porosity of 98%) irradiated up to a proton fluence of 5 × 10^14^ cm^−2^. The cross-section image can be divided into two areas: damage area (proton effect area) and a damage-free one (out of proton range). It can be seen that the cross-section morphologies of the proton-irradiated sample show distinct features between the damaged area and damage-free aera. The proton effect region is smoother and denser than the damage-free area, as reported in our previous work [28]. The average ranges calculated by GEANT4 and SRIM were 25.5 and 29.8 μm (as shown in Figure 6), respectively. Hence, the thickness of the damaged zone is about 22.7 μm, which is slightly smaller than the GEANT4 calculated result. This slight deviation should be attributed to the shrinkage of the aerogel during irradiation. However, there is a larger deviation to that simulated using SRIM code. It means that for a porous matter, there is some risk to evaluate the range or damaged behaviors with simulation methodology using the traditional SRIM code or density-homogenized equivalence modeling, and the higher the porosity is, the more risk for the evaluation process.

## 4. Conclusions

The transport behaviors of protons into nanoporous matter were investigated. The proton distribution moves to a deeper position and becomes wider as porosity increases, and the existence of voids causes the local inhomogeneity of proton distribution. Furthermore, the pore size affects the proton distribution, even though the porosity keeps same. The larger the void’s size, the deeper the proton average range is, and the broader the FWHM of the Bragg peak. Comparing the different simulation codes, GEANT4 code could be more suitable to simulate the particle transporting processes, thus, to evaluate the inhomogeneous interaction and energy deposition behaviors, and to determine precisely the particle range.

## Data Availability

The data that support the findings of this study are available from the corresponding author, [Yiyong Wu and Dandan Ju], upon reasonable request.
